# A Case of Inertia Uteri

**Published:** 1886-11

**Authors:** C. L. Gwyn

**Affiliations:** Galveston, Texas


### A CASE OF INERTIA UTERI.

*By C. L. Gwynn, M. D., Galveston, Texas.*

(For Daniel's Texas Medical Journal.)

ON the 5th of March 1880 I was called to Mrs. K., age 22 years a native of Alabama, married and a primipara. I found her suffering with general anasarca, and by her calculations within three weeks of her expected confinement.

The history of the case, as far as I could learn, was that she was chlorotic from the commencement of her menstrual life, and had lived for the past two years in the Brazos bottom, a very malarious district. She had suffered intensely from morning sickness from the beginning of her pregnancy to the present. I gave R. Cerii oxalat gr. ij. every second hour with some relief. I also gave her R. Pil Digitalis Comp every 4th hour with the result of giving tone and controlling the heart's action; there was also a slight increase in the amount of urine voided. I had no test case or apparatus for the examination of the urine for albumin, so cannot say whether it was present or not, or if present, in what quantities.

March 8th. The amount of the dropsy largely increased; the labia pudendi (which I had not examined before) enlarged to the size of small cocoanuts. For their relief I used acupuncture and gave fl. ext. Buchu in ʒ doses, also sub-carbonate of iron and nourishing diet. March 10th. Found to-day the labia almost normal in size; the dropsy of the lower extremities almost gone, and the patient feeling better. I discontinued the iron, as it produced gastric irritation.

March 11th. About day I was called to this case in labor, she had had slight pains since midnight; her mother had given her a half grain dose of morphine. Upon making a digital examination per vagina, I found the os tincæ barely dilated enough to insert the forefinger; the waters unruptured, the position of the child right lateral, with the spine to the front. According to her calculations she lacked some days of being at full term, and I determined to await the effect of the morphia; about noon labor recommenced, spontaneous version having taken place, the vertex presented. Owing to the anæmic condition of the patient it was slow and almost powerless, inertia taking place, and the patient being about to sink from exhaustion, having an intolerance to alcoholic stimulants, my forceps being at home six miles away. I applied five drops of nitrite of amyl by inhalation, with the immediate effect of increasing heart action and uterine contractions. I repeated the inhalation in fifteen minutes, with like effect, and again in fifteen minutes more with the effect of bringing the child and secundines into the world; there was less wasting or hemorrhage following this delivery than in any I had conducted for years.

The labor lasted, from the first pains, about fifteen hours. The baby was a well formed girl weighing about five pounds.

March 13th. Patient seemingly doing well, but as she does not rally in strength, I have ordered the baby to be taken away from her, and tonics and nutritive diet given.

March 17th. Patient gradually sinking in spite of tonics, restorative medicines, food, etc.

March 20th. Died to-day of anæmic exhaustion leaving the infant to follow her six months later, of infantile diarrhœa.

